# Supplementary material for: Copper Oxide Nanoparticles Induce Pulmonary Inflammation and Exacerbate Asthma via the TXNIP Signaling Pathway
Source: Int J Mol Sci. 2024 Oct 24;25(21):11436. doi: 10.3390/ijms252111436 (PMC11546552; doi:10.3390/ijms252111436)
Supplement: Supplementary file 1 [file ijms-25-11436-s001.zip › ijms-3217369-supplementary.pdf]

Copper Oxide Nanoparticles Induce Pulmonary Inflammation and Exacerbate Asthma via the TXNIP Signaling Pathway

Woong-Il Kim [1], So-Won Pak [1], Se-Jin Lee [1], Sin-Hyang Park [1], Je-Oh Lim [2], In-Sik Shin [1], Jong-Choon Kim [1,*] and Sung-Hwan Kim [3,*]

[1] College of Veterinary Medicine and BK21 FOUR Program, Chonnam National University, Gwangju 61186, Republic of Korea; dvmwoong@gmail.com (W.-I.K.); dvmpsw@gmail.com (S.-W.P.); xhdhksdl123@naver.com (S.-J.L.); shinhyang23@gmail.com (S.-H.P.); dvmmk79@gmail.com (I.-S.S.)

[2] Herbal Medicine Resources Research Center, Korea Institute of Oriental Medicine, Naju 58245, Republic of Korea; dvmljo@kiom.re.kr

[3] Jeonbuk Department of Inhalation Research, Korea Institute of Toxicology, Jeongup 56212, Republic of Korea

* Correspondence: toxkim@jnu.ac.kr (J.-C.K.); sunghwan.kim@kitox.re.kr (S.-H.K.); Tel.: +82-62-530-2827 (J.-C.K.); +82-63-570-8757 (S.-H.K.)

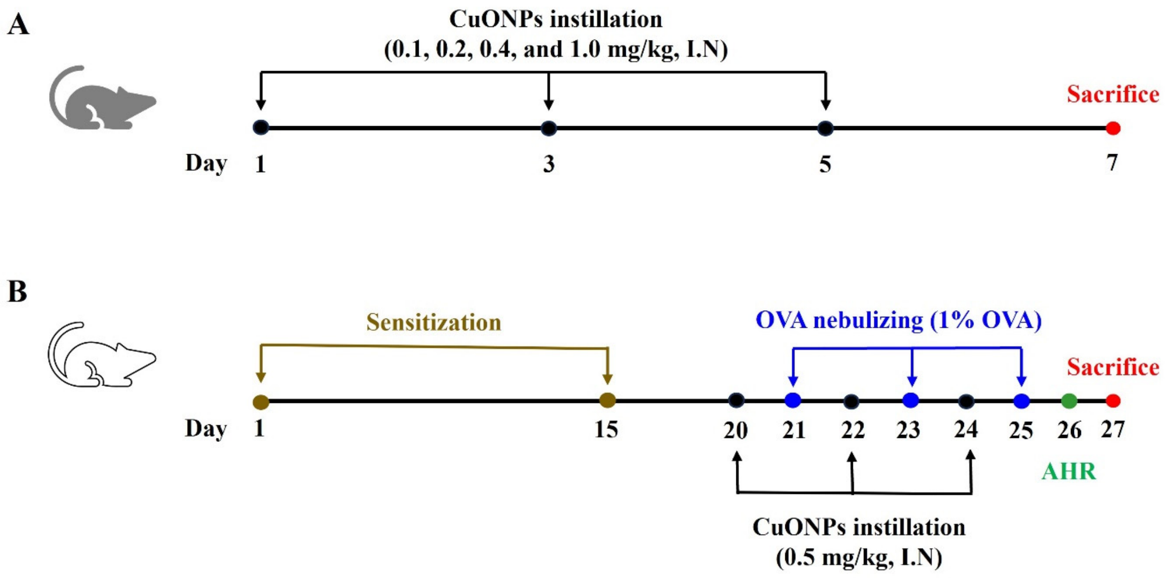

Figure S1. Illustration showing the experimental design. (**A**) CuONPs-induced pulmonary toxicity experimental schedule. (**B**) CuONPs-induced asthma exacerbation experimental schedule.

Table S1. Real-time PCR primer sequences.

Genes	Forward Primer (5′→3′)	Reverse Primer (5′→3′)
IL-1β	AGC CAG GAC AGT CAG CTC TC	ACT TCT TGC CCC CTT TGA AT
IL-6	ATG CAA TAA CCA CCC CTG AC	ATC TGA GGT GCC CAT GCT AC
TNF-α	CAA AGT AGA CCT GCC CAG AC	GAC CTC TCT CTA ATC AGC CC
GAPDH	CAA AAG GGT CAT CAT CTC TG	CCT GCT TCA CCA CCT TCT TG

IL-1β, interleukin-1β; IL-6, interleukin-6; TNF-α, tumor necrosis factor-alpha; and GAPDH, glyceraldehydes-3-phosphate dehydrogenase.
